# Neuronal activity controls transsynaptic geometry

**DOI:** 10.1038/srep22703

**Published:** 2016-03-08

**Authors:** Oleg O. Glebov, Susan Cox, Lawrence Humphreys, Juan Burrone

**Affiliations:** 1 Wolfson Centre for Age-Related Diseases, King’s College London, London SE1 1UL, UK; 2 Department of Developmental Neurobiology, King’s College London, London SE1 1UL, UK; 3 Randall Division of Cell and Molecular Biophysics, King’s College London, London SE1 1UL, UK

## Abstract

The neuronal synapse is comprised of several distinct zones, including presynaptic vesicle zone (SVZ), active zone (AZ) and postsynaptic density (PSD). While correct relative positioning of these zones is believed to be essential for synaptic function, the mechanisms controlling their mutual localization remain unexplored. Here, we employ high-throughput quantitative confocal imaging, super-resolution and electron microscopy to visualize organization of synaptic subdomains in hippocampal neurons. Silencing of neuronal activity leads to reversible reorganization of the synaptic geometry, resulting in a increased overlap between immunostained AZ and PSD markers; in contrast, the SVZ-AZ spatial coupling is decreased. Bayesian blinking and bleaching (3B) reconstruction reveals that the distance between the AZ-PSD distance is decreased by 30 nm, while electron microscopy shows that the width of the synaptic cleft is decreased by 1.1 nm. Our findings show that multiple aspects of synaptic geometry are dynamically controlled by neuronal activity and suggest mutual repositioning of synaptic components as a potential novel mechanism contributing to the homeostatic forms of synaptic plasticity.

The structure of the excitatory synapses comprising the central nervous system (CNS) is defined by the ordered progression of structurally and functionally distinct zones, including synaptic vesicles zone (SVZ), presynaptic active zone (AZ), synaptic cleft, and post-synaptic density (PSD)^1^. The prevailing view of the synaptic architecture is largely informed by the transmission electron microscopy (TEM) studies, suggesting the uniform and stable axial build-up of the synapse^2^,^3^. A recent study using super-resolution light microscopy, however, has revealed substantial variations in the localization of synaptic subdomain markers, hinting at the underlying diversity of synaptic architecture^4^. The potential importance of dynamic geometry for regulation of synaptic function has been explored in multiple theoretical studies^5^,^6^,^7^,^8^,^9^,^10^,^11^. However, to this day it is not known whether – and how – neuronal activity may be linked to the regulated distribution of synaptic domains.

To address this question, we have visualized how activity regulates distribution of the synaptic subdomains in hippocampal neurons. Our findings demonstrate that blockade of neuronal activity results in profound reversible remodelling of the synaptic structure, characterized by an increase in the AZ-PSD association and an increase in the AZ-SVZ association; furthermore, the distance between the presynaptic and postsynaptic membranes, *i.e.* the width of the synaptic cleft, is also decreased. These data reveal the large extent of activity control over the structure of the synapse and suggest a novel structural mechanism for regulation of synaptic function.

## Results

**Figure 1:**
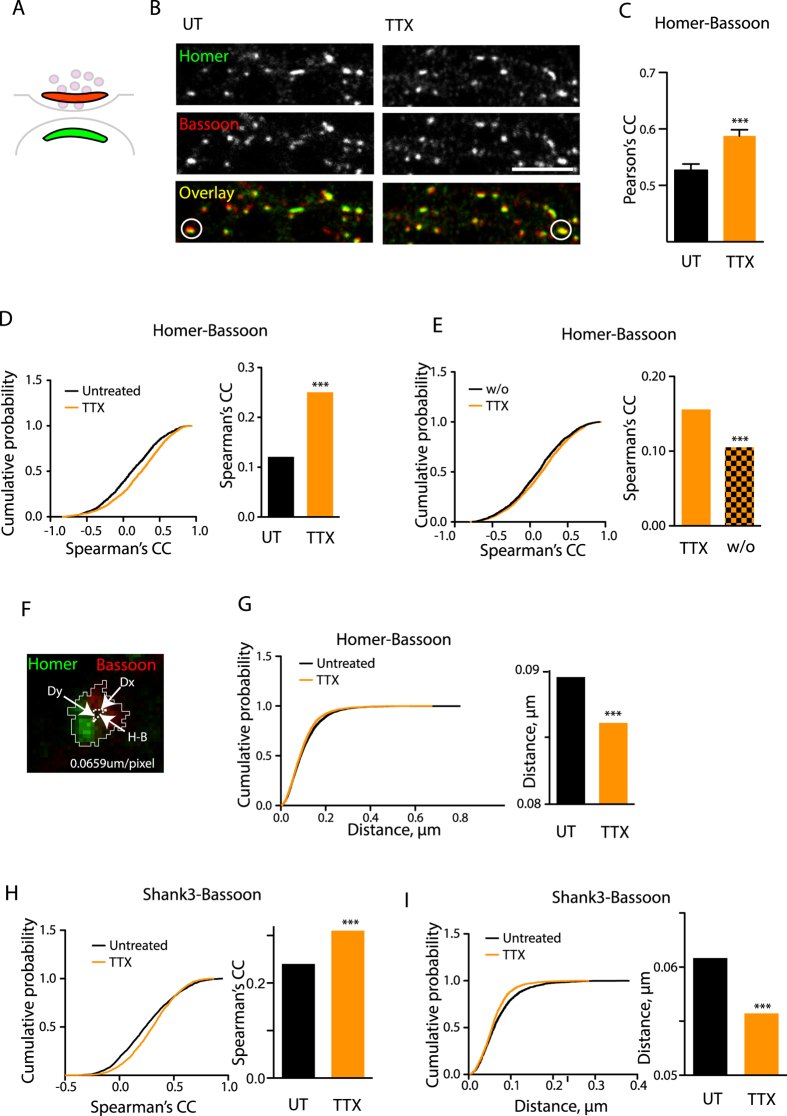
Blockade of neuronal firing by TTX leads to remodelling of the PSD-AZ architecture. (**A**) Schematic view of the synapse with the relevant domains highlighted. Green, PSD (Homer); red, AZ (Bassoon). (**B**) Staining for Bassoon and Homer in control or TTX-treated (2M, 48 h) neurons. The representative puncta with overlapping Homer and Bassoon are encircled. (**C**) Colocalization between Homer and Bassoon in untreated and TTX-treated neurons based on whole fields of view. Shown are the mean values ±standard error from the mean (SEM). ***P < 0.0001, two-tailed Student t-test. N = 6 experiments. (**D**) Cumulative probability plot and median values for the Spearman’s correlation coefficients representing synapse-specific Homer-Bassoon colocalization. ***P < 0.0001, Mann-Whitney test. N = 4, n = 5033 (UT) and 4971 (TTX). (**E**) Cumulative probability plot and median values for Pearson’s correlation coefficients between Homer and Bassoon in TTX-treated cultures and sister cultures following the washout (w/o) of TTX (48 h). ***P < 0.0001, Mann-Whitney test. N = 3, n = 2601 (TTX) and 2067 (w/o). (**F**) Schematic representation of the method for calculating the distance across the synapse. Neurons were stained for Homer and Bassoon. Dx, Dy and Homer-Bassoon (H-B) distance form the two catheti and the hypotenuse respectively of the right-angled triangle. Dx and Dy are measured directly, and TSD is then calculated using the Pythagorean theorem. (**G**) Cumulative probability plot and median values for synapse-specific Homer-Bassoon distances. ***P < 0.0001, Mann-Whitney test. N = 4, n = 5021 (UT) and 5128 (TTX). (**H**) As in (**D**) but for Shank3-Bassoon pair. ***P < 0.0001, Mann-Whitney test. N = 3, n = 2160 (UT) and 2040 (TTX). **(I)** as in (**G**) but for the Shank3-Bassoon pair. ***P < 0.0001, Mann-Whitney test. N = 3, n = 2141 (UT) and 1671 (TTX). Scale bar, 10m.

To address the hypothesis that trans-synaptic geometry may be subject to regulation by neuronal activity, we applied a blocker of voltage-gated sodium channels tetrodotoxin (TTX) for 48 h and visualized the distribution of the canonical PSD and AZ markers Homer and Bassoon (Fig. 1A,B). In contrast to the cortical neurons^12^, activity blockade did not result in a decreased abundance of either Homer or Bassoon and did not affect the synaptic area (Supplementary Fig. S1A), but instead increased their levels by 28.8% and 22.1% respectively (Fig. 1B, Supplementary Fig. S1B,C), highlighting the fundamental differences in activity-dependent plasticity between cortical and hippocampal synapse.

To assess whether the synaptic geometry was changed by activity blockade, we quantified the extent of colocalization between Bassoon and Homer either in whole images or synapse-specifically, reasoning that alterations in synaptic geometry may manifest themselves through alterations in the correlation between immunofluorescence intensities on a pixel-by-pixel basis. Indeed, TTX treatment reversibly increased the overall and the synapse-specific Homer-Bassoon colocalization (Fig. 1B–E, Supplementary Fig. S1D). This effect was observed after 24 h but not after 1 h (data not shown), suggesting that the timescale and the mechanisms underlying this phenomenon were distinct from the previously reported rapid structural plasticity of PSD and AZ^13^,^14^,^15^,^16^. Despite the increase in accumulation of Homer and Bassoon (Supplementary Fig. 1B,C), the TTX-induced increase in Homer-Bassoon colocalization was independent of the levels of Homer or Bassoon or synaptic area (Supplementary Fig. S1E–G), indicating that the observed effect was likely due to the structural changes affecting the relative positioning of AZ and PSD within the synapse rather than an alteration in their compactness or size.

To obtain a more direct measurement of the structural alterations within the synapse, we measured the effect of TTX on the geometrical distance between the centres-of-mass of the adjacent Bassoon- and Homer-positive clusters (Fig. 1F). In agreement with the colocalization data, TTX treatment reduced this distance (Fig. 1G). The distance was consistently lower in the TTX-treated cultures irrespective of the synaptic area or the levels of either Homer or Bassoon (Supplementary Fig. 2A–C), further confirming that the changes in the trans-synaptic geometry were uncoupled from recruitment of synaptic machinery. Similar effects were observed for the overlap between another PSD marker Shank3 and Bassoon (Fig. 1H,I, Supplementary Fig. S3).

**Figure 2:**
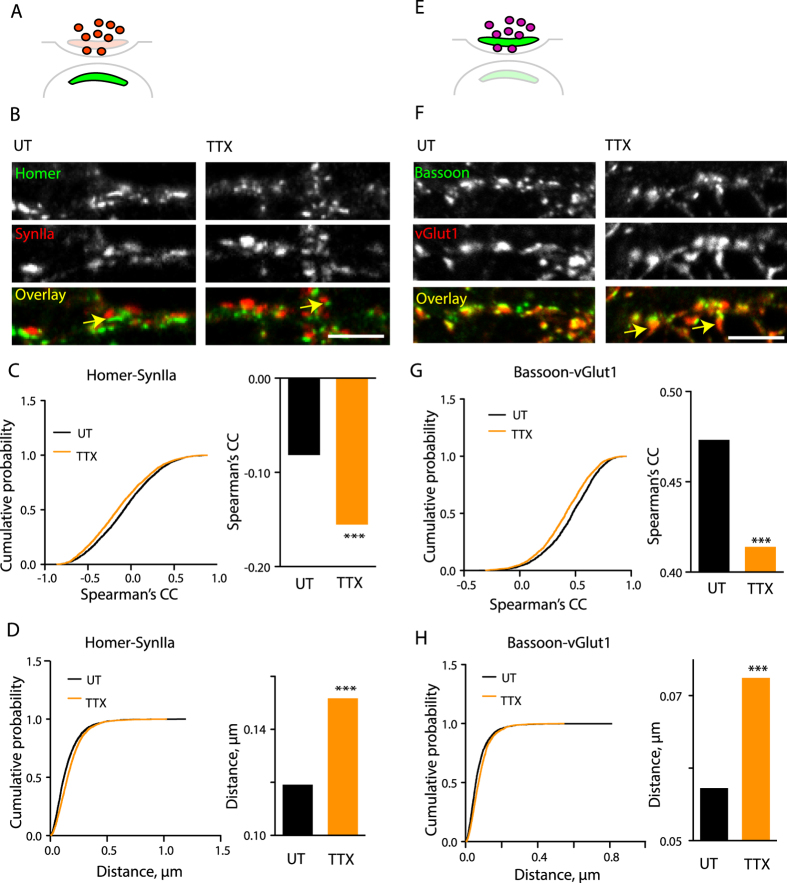
Blockade of neuronal firing by TTX leads to remodelling of the AZ-SVZ and PSD-SVZ architecture. (**A**) Schematic view of the synapse with the PSD and SVZ domains highlighted. Green, PSD (Homer); red, SVZ (SynIIa). (**B**) Staining for Homer and SynIIa in control or TTX-treated neurons. Arrows denote the visible gaps between apposed Homer- and SynIIa-positive clusters. **(C)** Cumulative probability plot and median values for Spearman’s correlation coefficients representing synapse-specific Homer-SynIIa colocalization. ***P < 0.0001, Mann-Whitney test. N = 3, n = 2575 (UT) and 2954 (TTX). (**D**) Cumulative probability plot and median values for synapse-specific Homer-SynIIa distances. ***P < 0.0001, Mann-Whitney test. N and n, see above. **(E)** as in (**A**) but for AZ and SVZ. Green, AZ (Bassoon); red, SVZ (vGlut1). (**F**) as in (**B**) but for the Bassoon-vGlut1 pair. Arrows denote the areas of SVZ extending away from the corresponding AZ. (**G**) as in (**C**) but for the Bassoon-vGlut1 pair. ***P < 0.0001, Mann-Whitney test. N = 3, n = 2323 (UT) and 2204 (TTX). (**H**) as in D but for the Bassoon-vGlut1 pair. N and n, see above. ***P < 0.0001, Mann-Whitney test. N = 3. Scale bar, 5m.

To test the effect of TTX treatment on the mutual distribution of the PSD and SVZ, we labelled neurons for Homer and an SVZ marker Synapsin IIa (SynIIa) (Fig. 2A). In agreement with their targeting to the opposite synaptic sides, there was little overlap between the Homer and the SynIIa-positive puncta (Fig. 2B), a gap between Homer- and SynIIa-positive clusters was often visible and synapse-specific Homer-SynIIa colocalization was negative, indicative of a mutual spatial exclusion between the SVZ and the PSD (Fig. 2B,C). SynIIa-Homer colocalization was further decreased by the TTX treatment (Fig. 2B,C), consistent with increased spatial separation between SVZ and PSD, while the distance between the centres of SynIIa and Homer-positive clusters was increased accordingly (Fig. 2D). We therefore conclude that neuronal activity regulates the distance between PSD and SVZ in an opposite direction to that of PSD and AZ.

To test the effect of TTX treatment on the mutual distribution of the AZ and SVZ, we visualized Bassoon and another SVZ marker vGlut1. In contrast to Homer and SynIIa, these two markers showed considerable overlap, indicative of a close spatial association between the AZ and the SVZ (Fig. 2E,F). Treatment with TTX, however, led to a substantial part of the vGlut1-positive clusters extending away from the AZ and a corresponding decrease in the Bassoon-vGlut1 colocalization (Fig. 2G,F), while the Bassoon-vGlut1 distance was increased (Fig. 2H). Thus, blockade of neuronal activity disrupts the tight association between the AZ and SVZ domains.

To establish the postsynaptic signalling pathway involved in controls of synaptic geometry, we pharmacologically blocked the activity of the three major classes of receptors involved in glutamatergic synaptic transmission. While blockade of AMPA and kainate-type receptor by 2,3-dihydroxy-6-nitro-7-sulphamoyl-benzo(f)quinoxaline-2,3-dione (NBQX) did not affect Homer-Bassoon the overlap (Supplementary Fig. S4A), application of the NMDA-type receptor (NMDAR) antagonist (2R)-amino-5-phosphonovaleric acid (APV) and a different NMDAR antagonist MK-801 increased Bassoon-Homer colocalization and decreased Bassoon-Homer distance (Supplementary Fig. S4B–D). These data suggest that NMDAR activity is necessary for maintenance of synaptic architecture.

**Figure 3:**
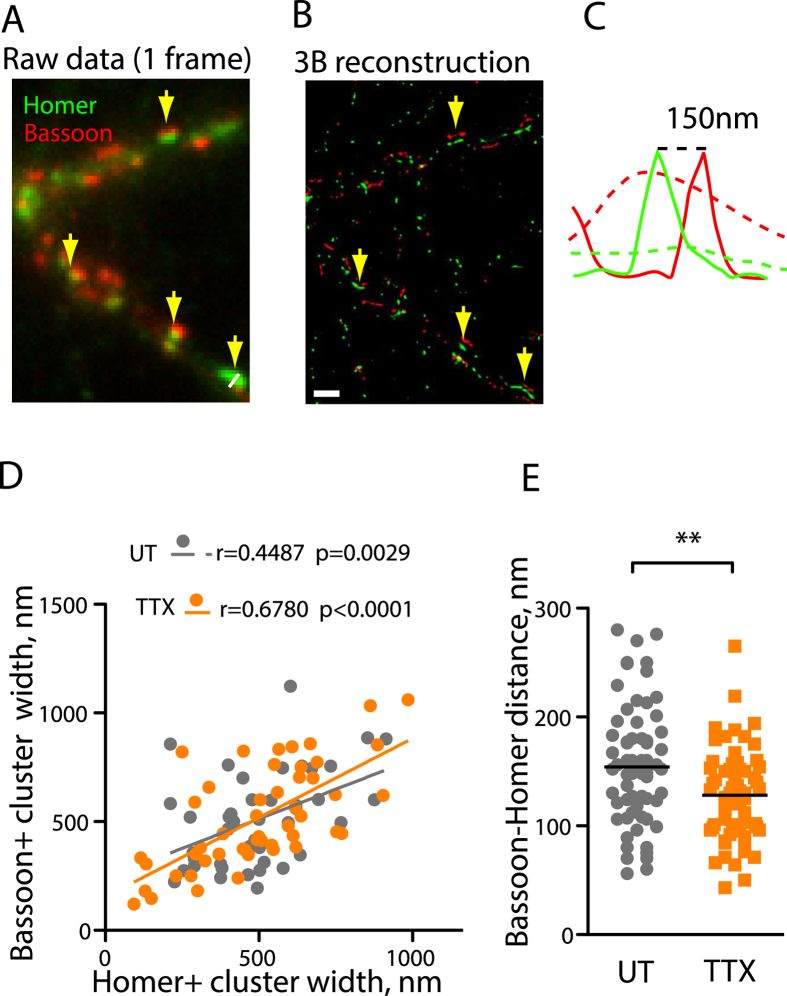
Super-resolution 3B imaging of TTX-induced trans-synaptic remodelling. (**A**) A representative image of one frame used for 3B reconstruction. Homer, green; Bassoon, red. Arrows denote the regions of Bassoon-Homer overlap (**B**) 3B reconstruction of (**A**) (>200 frames). Regions denoted by arrows manifest characteristic morphology of excitatory synapses with Bassoon-positive AZ and Homer-positive PSD separated by a clear gap. (**C**) Bassoon and Homer Intensity profiles along the line in (**A**) (dashed lines) and (**B**) (solid lines). (**D**) Correlation plot between AZ and PSD width in individual synapses. Pearson’s coeffficient was used for quantifying correlation. (**E**) TTX treatment resulted in a decreased Bassoon-Homer distance. Horizontal lines indicate mean values. **P = 0.001, two-tailed Student t-test, N = 3, n = 43 (UT) and 49 (TTX). Scale bar, 3m.

To directly measure the nanoscale alterations in synaptic structure, we employed a recently developed super-resolution 3B (Bayesian Blinking and Bleaching) method which was previously shown to achieve a spatial resolution of 50 nm^17^. In contrast to conventional microscopy, application of 3B imaging in a near-TIRF mode allowed us to obtain a clear separation between AZ and PSD and directly quantify the geometry of the individual synapses (Fig. 3A–C). The Bassoon-Homer distance measured using 3B amounted to 164.8 + −8.9 nm, which is comparable to a value obtained in a previous superresolution study in brain sections^4^. In the lateral dimension, the AZ and PSD lengths were more variable, but as reported before^2^ there was a strong synapse-specific correlation between the width of the AZ and PSD; interestingly, this correlation was unchanged by the TTX treatment, indicative of the activity-independent nature of the AZ-PSD matching (Fig. 3D). In contrast to that, TTX treatment led to a reduction of the Homer-Bassoon distance to 132.0 + −6.3 nm (Fig. 3E), while having no effect on the width of the apposed AZ and PSD (Fig. 3D). This data directly confirm the evidence from confocal imaging demonstrating that activity blockade results in a nanoscale rearrangement of the transsynaptic architecture (Fig. 1).

**Figure 4:**
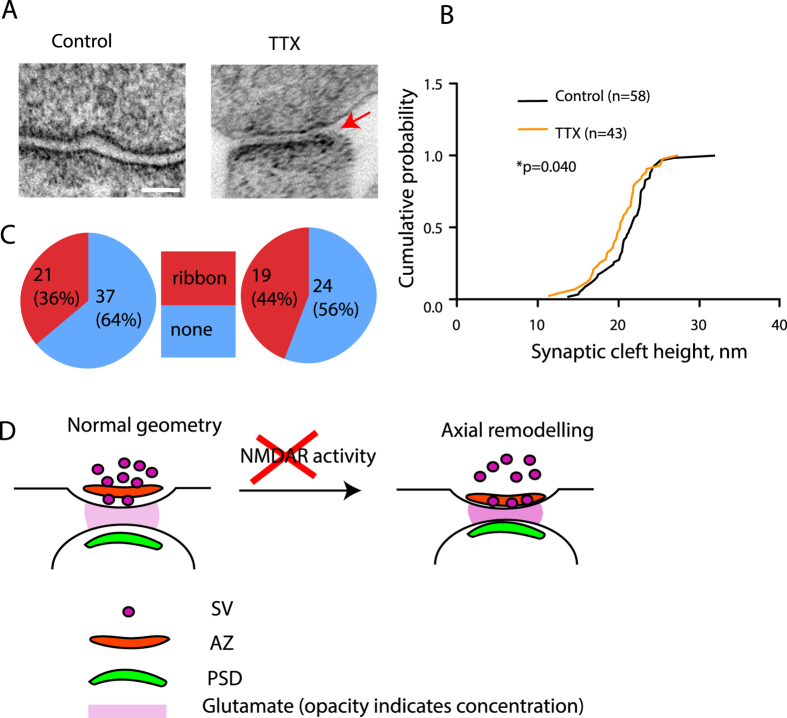
TEM imaging of the synaptic cleft width. (**A**) Representative images of the synapses from control and TTX-treated cultures. Arrow denotes electron-dense ribbon within the cleft. Scale bar, 50 nm. (**B**) Quantification of the effect of TTX on synaptic cleft width, p = 0.040, Kolmogorov-Smirnov test. N = 3, n = 58 (UT) and 43 (TTX). (**C**) Percentage of synapses exhibiting intracleft ribbons. (**D**) Proposed model of activity-dependent regulation of trans-synaptic geometry. At normal levels of activity, a degree of spatial separation is maintained between AZ and PSD and between the two sides of the synaptic cleft, while SVZ is associated with the AZ. Blockade of neuronal activity (likely that of NMDA receptors, see Fig. S4) results in a rearrangement of the synapse, leading to a decreased AZ-PSD distance; this coincides with a spatial SV-AZ uncoupling.

Finally, we decided to check whether the width of the synaptic cleft was also subject to regulation by activity. Computational studies posit that changes in the width of the synaptic cleft are likely to lead to alteration in synapse function^5^,^6^,^9^,^10^. As the small width of the cleft (20 nm) puts it beyond the reach of light microscopy, we used TEM to visualize the synaptic cleft in untreated and TTX-treated neurons, focusing on regions possessing the morphological hallmarks of excitatory glutamatergic synapses. Of note, TEM with glutaraldehyde fixation has been recently used to reveal 1–2 nm changes in synaptic cleft width, indicating that this protocol offers appropriate sensitivity to measure subtle alterations of synaptic geometry^18^. Untreated cultures had synapses with median cleft width of 21.22 nm, in good agreement with the literature^2^,^18^ (Fig. 4A). Treatment with TTX however reduced the synaptic cleft width (median value 20.14 nm, p = 0.040) (Fig. 4B). Interestingly, a ribbon-like accumulation of electron-dense material in the cleft (Fig. 4A, arrow) was present in 36% of control synapses and 44% of TTX-treated synapses (Fig. 4C). Thus, blockade of neuronal firing not only leads to the remodelling of the AZ and the PSD, but also affects the geometry of the synaptic cleft.

## Discussion

We have used several imaging approaches to interrogate the relationship between neuronal activity and synaptic geometry. Our data demonstrates that cessation of neuronal firing induces spatial remodelling of the functional zones across the synapse (Fig. S5). The notion of trans-synaptic rearrangement is further supported by both super-resolution light microscopy (Fig. 3) and EM (Fig. 4), which respectively show a TTX-induced rearrangement of the Homer- and Bassoon-positive zones and a decrease in the width of the synaptic cleft. The lateral distribution of synaptic markers, on the other hand, appears to be preserved, although it remains possible that the nanoscale changes or topological alterations may occur within the synaptic domains below resolution of super-resolution imaging^19^,^20^ and may contribute towards the observed changes in colocalization.

The large decrease in the distance between the centres of the Homer and Bassoon clusters (circa 40 nm) is in contrast with the relatively minor 1.1 nm decrease in the synaptic cleft width, suggesting that the decrease in the distance between the pre-and post-synaptic membrane may be accompanied by the axial redistribution of the material within the AZ and/or the PSD domains. Furthermore, 3B imaging in the near-TIRF mode does not take into account the three-dimensional orientation of the synapse, and thus the TSD distance measured using this approach is likely to be an underestimate. Considering the multiple steps and potential artefacts involved in the sample preparation of brain sections for either super-resolution^4^ or electron microscopy^21^, rigorous comparative studies in cultures, brain slices and sections will be highly advantageous for directly addressing the activity-dependent ultrastructural dynamics of the synaptic geometry.

Our finding expands the repertoire of potential mechanisms for homeostatic control of synaptic functionality^22^ and provides support for the previously suggested link between activity, synaptic geometry and function^5^,^6^,^8^,^9^,^10^,^11^. On this basis, we propose a model for homeostatic activity-dependent regulation of synaptic geometry (Fig. 4D), whereby ongoing neuronal activity maintains the synaptic geometry, and activity blockade triggers reversible mutual repositioning of synaptic functional zones. The molecular basis for the trans-synaptic link between activity and structure remains to be ascertained; in particular, it will be fascinating to test for the potential involvement of activity-dependent modulation of trans-synaptically interacting proteins such as neuroligins^23^,^24^ and neurexins^25^ which are associated with the electron-dense accumulations in the synaptic cleft^26^ (Fig. 4).

How does this structural alteration translate to a functional change? The current debate over the identity and localization of the distinct vesicle pools^27^,^28^,^29^,^30^ precludes adequate interpretation of the observed changes in the SVZ, although it is tempting to speculate that the observed remodelling may be associated with the inactivity-induced changes in the SVZ structure and dynamics^31^,^32^,^33^. On the other hand, the decreased width of the synaptic cleft is likely to affect the glutamate concentration and kinetics of glutamate clearance, in agreement with previous theoretical studies^6^,^10^,^34^ and experimentally reported inactivity-induced increase in intra-cleft glutamate concentration^35^. The geometrical effect is likely to be further enhanced by the non-linear relationship between postsynaptic current and glutamate concentration^36^, spatiotemporal profile of synaptic glutamate concentration^34^,^37^, and non-random distribution of synaptic machinery on the nanoscale^19^,^38^,^39^.

Our results suggest that TTX treatment results not only in the increased proximity of the PSD and AZ is defined by clustering of Homer and Bassoon (Fig. 3), but also in an increase in the synaptic recruitment of these two proteins, in contrast with a previous study using cortical neurons^12^. This discrepancy can be explained by the difference in experimental system, highlighting the potential diversity of the synaptic mechanisms underlying homeostatic plasticity across the CNS. Nevertheless, the increase in synapse-specific trans-synaptic association was observed in all synapses irrespective of their area or levels of synaptic markers (Supplementary Figs S1 and S2), indicating that the factors other than the increase in synaptic recruitment account for the observed changes.

The nanoscale organization of the synapse is believed to be critical for its function, as multiple functional elements within the synapse must be in sufficient spatial proximity to allow for synaptic transmission^40^,^41^. Within this framework, reversible repositioning of synaptic subdomains provides a simple and cost-effective way for the neuron to allow for homeostatic modulation of synaptic strength. Interestingly, our results echo early morphological observations in other experimental systems^42^,^43^, suggesting that this form of structural plasticity may represent an evolutionarily conserved phenomenon. Given the ongoing controversy surrounding the mechanisms of Hebbian forms of plasticity such as LTP^44^, it will be fascinating to see whether structural synaptic remodelling may be involved in these non-homeostatic input-specific forms of plasticity. The relationship between synaptic geometry and function and the molecular mechanisms linking these two properties will therefore warrant future investigation.

## Materials and Methods

### Reagents

Cell culture media was from Invitrogen. Poly-L-lysine was from Sigma. The following primary antibodies were used: mouse monoclonal anti-Bassoon clone SAP7F407 (Abcam, UK), mouse monoclonal anti-Synapsin IIa (BD Biosciences, UK), rabbit polyclonal anti-Homer, rabbit polyclonal anti-Shank3, rabbit polyclonal anti-vGlut1 (Synaptic Systems, Germany). Secondary antibodies were from Jackson Immunoresearch (USA). APV, NBQX, TTX were from Tocris (UK).

### Cell culture

Dissociated hippocampal neuronal cultures were prepared from E18 rat embryos and cultured according to the Banker protocol. All experiments involving neurons were carried out at 16–21 days *in vitro*. For confocal imaging, cells were plated onto 13 mm round glass coverslips (thickness 1.0) placed in 35 mm Petri dishes (4/dish). For 3B imaging, cell were plated onto either 22 mm square glass coverslips (Carl Zeiss, Germany) or 35 mm μ-Dish (Ibidi, Germany), both thickness 1.5. To reduce variability, each experiment was carried out using sister cultures, i.e. cultures originating from the same preparation and cultured identically; to further enhance reproducibility, each confocal imaging experiment was carried out using the coverslips cultured within the same Petri dish.

### Confocal microscopy

After treatment, coverslips were fixed with 4%PFA in PBS for 15–20 min at room temperature (RT) and permeabilized in 0.3%Triton-X100 in PBS supplemented with 5% horse serum for 10 min. Subsequent incubations were carried out in the permeabilization buffer. Coverslips were incubated with appropriate primary antibodies for 60 min at RT, washed 4 times in PBS and incubated with AlexaFluor-488, AlexaFluor568- and AlexaFluor647-conjugated secondary antibodies at a concentration of 0.3 ug/ml each for 60 min at RT. Coverslips were then mounted in mounting medium (Southern) and imaged on a Zeiss LSM710 microscope equipped with a standard set of lasers through a 63× oil objective. The imaging system was controlled by the ZEN software. To minimize observational bias, regions of interest were chosen by zooming on one channel (typically showing Bassoon or SynapsinIIa fluorescence) blind with respect to the other channel. Regions of interest sized 1024 × 1024 pixels (65.8 nm/pixel) were imaged at speed 7 with the averaging setting 2. We note that the effect of the experimental treatment was robust against variations in acquisition settings such as doubling the pixel size and increasing the scanning speed. Excitation wavelengths were 488, 543 nm and 633 nm. Bandpass filters were set at 500–550 (AlexaFluor488), 560–615 nm (Cy3, AlexaFluor568) and 650–750 nm (AlexaFluor647). Image acquisition was carried out at the 12-bit rate. Settings were optimized to ensure appropriate dynamic range, low background and sufficient signal/noise ratio.

### Colocalization analysis

Colocalization was determined using ImageJ plugins “Colocalization Threshold” for whole images and “Coloc2” for individual synapses. For whole images, Pearson’s correlation coefficient was quantified; for individual synapses, Pearson’s correlation coefficient or Spearman’s correlation coefficient was used. To identify individual synapses, images were binarized in ImageJ using the “Moments” setting, and particles were counted automatically using the “Analyze Particles” command across the whole image excluding the cell body. Binarized data from either Homer or Bassoon channels was used for determination of synapses; alternatively, areas containing adjacent Homer and Bassoon puncta were collated using Image Math function with Add setting, and resulting ROIs were identified using the Add Particles function. To minimize contamination of the data with ROIs arising from non-specific staining or overlap of multiple synapses, only ROIs with areas ranging from 0.2 to 3 μm^2^ were included in further analysis. All values of circularity were included in analysis. Since background fluorescence intensity was typically less than 2% of the median ROI fluorescence, background subtraction did not significantly affect the colocalization measurements and was not performed.

### Synapse-specific analysis of geometric distance

Quantitative data pertaining to the individual synapses was extracted using ROI Manager. Pearson’s Correlation Coefficients for colocalization were calculated using the Coloc2 plugin. Area, mean intensity, positions of the centres-of-mass were calculated using Measure function for each channel in each synapse. The TSD value was derived from the positions of the centers of mass using the Pythagorean theorem:


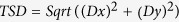


where Dx and Dy are distances between the presynaptic and postsynaptic markers’s centers of mass along the two axes of the 2D image.

### 3B imaging

The detailed protocol for this method can be found elsewhere^17^. Briefly, coverslips were processed as for confocal microscopy, except that the secondary antibodies were used at 1 ug/ml and the anti-mouse secondary antibody was conjugated to DyLight649. Samples were then incubated in the STORM imaging buffer with MEA (for recipe see http://www.nikoninstruments.com/en_GB/Products/Microscope-Systems/Inverted-Microscopes/N-STORM-Super-Resolution/(brochure)) in the following manner. The 22 mm square coverslips were lifted from their dishes, excess buffer was removed by blotting with paper and the coverslips were upturned onto 30 μl of the imaging buffer placed onto a glass slide. Coverslips were secured on the slide using Vaseline, and excess imaging buffer was blotted out with paper. With glass-bottomed dishes, 300 μl imaging buffer was added directly onto the dishes. In both cases, imaging was performed immediately after mounting. Imaging was performed on a Nikon Ti-E TIRF inverted microscope equipped with an Andor iXon DU897 EMCCD camera and a Perfect Focus system, running on the NIS Elements software. For optimal signal/noise ratio, near-TIRF angle was used. Imaging was carried out at the 12-bit rate through a 100× oil objective with a 488 nm and a 647 nm laser. ROI was set at 128 × 128 pixels, with the pixel size of 160 nm. Image acquisition was performed at 50 Hz, sequentially for AlexaFluor488 and DyLight649. Four laser power settings (10%, 30%, 60% and 100%) were manually triggered to ensure the optimal blinking and bleaching properties of the fluorophores. Image acquisition was allowed to proceed for at least 400 frames at each laser power setting in order to reach a number of frames sufficient for super-resolved reconstruction by the 3B algorithm. The data was exported in the TIFF format. Masks of the images were made by hand, to identify those areas of the image in which synapses occurred. The image was then split into areas of 15 × 15 pixels and the 3B algorithm was run, with the run for each area using single core of a cluster computer. The pixel size was 160 nm and the point spread function size was estimated to be 300 nm. After a minimum of 200 iterations per area the data was collected and a super-resolution probability map of the image was reconstructed. The reconstructed image, a probability density map of fluorophore positions, had a pixel size of 10 nm and a point spread function size of 20 nm.

### Transmission electron microscopy

The sister cultures were either left untreated or treated with 2M TTX for 48 hours. Cultures were then fixed in 2.5% glutaraldehyde (pH 7.3), post-fixed in osmium tetroxide fixative (Millonig’s buffer, pH 7.3) for 90 min at 4 °C and embedded in TAAB resin. Ultra thin sections of the selected viewing area (60 nm thickness) were cut on Leica Ultra-cut machines and picked up on to 150 mesh Guilder grids with a support film of Pioloform. The grids were stained with uranyl acetate and lead citrate. Images were acquired using the Hitachi H&600 Transmission Electron Microscope at 70000× magnification. For identification of the excitatory synapses, we have focused on the morphological hallmarks of such synapses described previously, namely asymmetric distribution of vesicles and a prominent electron-dense PSD. These properties are commonly accepted as reliable markers of excitatory synapses both in culture and *in vivo*^45^. We ensured that the length of the AZ/PSD in selected synapses was less than 800 nm, as this is the maximal reported length of the synapse in cultured neurons^2^. As long as this requirement was met, unequivocally asymmetric synapses were considered irrespective of their putative segmentation. An average of three measurements of synaptic cleft width evenly spaced across the synapse was taken as a reading for each synapse, where the ends of the synapse were defined as the ends of the electron-dense PSD. There was no significant difference between the widths measured at different positions of the synapse (data not shown). To avoid bias, images were analyzed blind to the experimental condition.

### Statistical analysis

All the experiments were performed at least in three independent biological replicates, with three fields of view per condition selected for analysis (see section on confocal microscopy). All of the synapses automatically detected within these fields of view were included in the analysis. Power analysis was used to confirm that the sample size was sufficient to detect the statistically significant differences in the median values. N and n correspond to the number of independent replicates and number of synapses respectively. Statistical analysis was carried out using the Prism 5.0 c software package (GraphPad Software). Data distributions were assessed for normality using d’Agostino and Pearson omnibus normality test. For normally distributed data, Student’s t-test and Pearson’s coefficient were quantified , otherwise Mann-Whitney test and Spearman’s coefficient were employed.

## Additional Information

**How to cite this article**: Glebov, O. O. *et al*. Neuronal activity controls transsynaptic geometry. *Sci. Rep.* **6**, 22703; doi: 10.1038/srep22703 (2016).

## Supplementary Material

Supplementary Information
