# Corrigendum: Neuronal activity controls transsynaptic geometry

**DOI:** 10.1038/srep26422

**Published:** 2016-05-31

**Authors:** Oleg O. Glebov, Susan Cox, Lawrence Humphreys, Juan Burrone

Scientific Reports 6: Article number: 2270310.1038/srep22703; published online: 03 08 2016; updated: 05 31 2016

This Article contains errors.

In the Results section,

“In contrast to the cortical neurons^12^, activity blockade did not result in a decreased abundance of either Homer or Bassoon and did not affect the synaptic area (Supplementary Fig. S1A), but instead increased their levels by 28.8% and 22.1% respectively (Fig. 1B, Supplementary Fig. S1B,C),”

should read:

“In contrast to the cortical neurons^12^, activity blockade did not result in a decreased abundance of either Homer or Bassoon and did not affect the synaptic area as defined by the Homer punctate staining (Supplementary Fig. S1A), but instead increased their levels by 28.8% and 22.1% respectively within the Homer-positive puncta (Fig. 1B, Supplementary Fig. S1B,C),”

In the legend of Supplementary Figure 1,

“**Supplementary Figure 1. Effect of activity blockade on synaptic area, levels of Homer and Bassoon, Homer-Bassoon colocalization values in three independent experiments, and the relationship between the colocalization values and synaptic properties. (A)** TTX treatment does not affect the size of synaptic clusters. P > 0.05, Mann-Whitney test. **(B)** TTX treatment increases synaptic levels of Homer. P < 0.0001, Mann-Whitney test. **(C)** TTX treatment increases synaptic levels of Homer. P < 0.0001, Mann-Whitney test.”

should read:

“**Supplementary Figure 1. Effect of activity blockade on synaptic area, levels of Homer and Bassoon within the Homer-positive puncta, Homer-Bassoon colocalization values in three independent experiments, and the relationship between the colocalization values and synaptic properties.** (A) TTX treatment does not affect the size of synaptic clusters. P > 0.05, Mann-Whitney test. (B) TTX treatment increases levels of Homer within the Homer-positive puncta. P < 0.0001, Mann-Whitney test. (C) TTX treatment increases levels of Bassoon within the Homer-positive puncta. P < 0.0001, Mann-Whitney test.”

