# Supplementary material for: Neuronal activity controls transsynaptic geometry
Source: Sci Rep. 2016 Mar 8;6:22703. doi: 10.1038/srep22703 (PMC4782104; doi:10.1038/srep22703)

Supplementary Information

Manuscript title: "Neuronal activity controls transsynaptic geometry"

Authors: Oleg O. Glebov, Susan Cox, Lawrence Humphreys and Juan Burrone

Supplementary Figure Legends

Supplementary Figure 1. Effect of activity blockade on synaptic area, levels of Homer and Bassoon, Homer-Bassoon colocalization values in three independent experiments, and the relationship between the colocalization values and synaptic properties. (A) TTX treatment does not affect the size of synaptic clusters. P>0.05, Mann-Whitney test. **(B)** TTX treatment increases synaptic levels of Homer. P<0.0001, Mann-Whitney test. **(C)** TTX treatment increases synaptic levels of Homer. P<0.0001, Mann-Whitney test. **(D)** TTX treatment significantly increased the Homer-Bassoon synapse-specific colocalization in three separate experiments. **(E-G)** The synapse-specific colocalization was consistently higher in TTX-treated cultures irrespective of the synaptic area (E), synapse-specific levels of Homer (F) and Bassoon (G). Locally weighted scatterplot smoothing (LOWESS) regression plots are shown. N=3, n=1729 (UT) and 1711 (TTX).

Supplementary Figure 2. The relationship between the TSD values and synaptic properties. The TSD values were consistently higher in the TTX-treated cultures irrespective of the synaptic area (A), synapse-specific levels of Homer (B) and Bassoon (C). Locally weighted scatterplot smoothing (LOWESS) regression plots are shown. N=3, n=1729 (UT) and 1711 (TTX).

Supplementary Figure 3. TTX treatment significantly alters the synapse-specific colocalization of synaptic markers in three separate experiments. (A) Individual experiments for Figure 1H. Expt1: n=438 (UT) and 415 (TTX), Expt 2: n=708 (UT) and 687 (TTX), Expt 3: n=1014 (UT) and 938 (TTX). **(B)** Individual experiments for Figure 2C. Expt1: n=1042 (UT) and 1144 (TTX), Expt 2: n=904 (UT) and 889 (TTX), Expt3: n=629 (UT) and 921 (TTX). **(C)** Individual experiments for Figure 2G. Expt1: n=653 (UT) and 498 (TTX), Expt 2: n=911 (UT) and 1246 (TTX), Expt 3: n=759 (UT) and 460 (TTX). *P<0.05, **P<0.001, ***P<0.0001, Mann-Whitney test.

Supplementary Figure 4. Distinct postsynaptic regulation of synaptic geometry. (A) NBQX (100uM, 48h) treatment has no effect on Homer-Bassoon overlap. N=5, n=2103 (UT) and 1368 (TTX). P>0.05, Mann-Whitney test. (**B)** APV (50nM, 48h) increases the Homer-Bassoon overlap N=3, n=2272 (UT) and 2377 (TTX). **(C)** APV decreases the Homer-Bassoon geometric distance. N and n, see above. **(D)** MK-801 (50uM, 48h) increases the Homer-Bassoon overlap. N=4, n=2571 (UT) and 2139 (MK-801).

Supplementary Figure 5. Graphic summary. (A) A schematic view of the synapse denoting the synaptic vesicles (SVZ), the active zone (AZ), and the postsynaptic density (PSD). Presynaptic and postsynaptic membranes are shown. **(B)** Summary of the experimental findings with references to the relevant figures.

Supplementary Figure 1

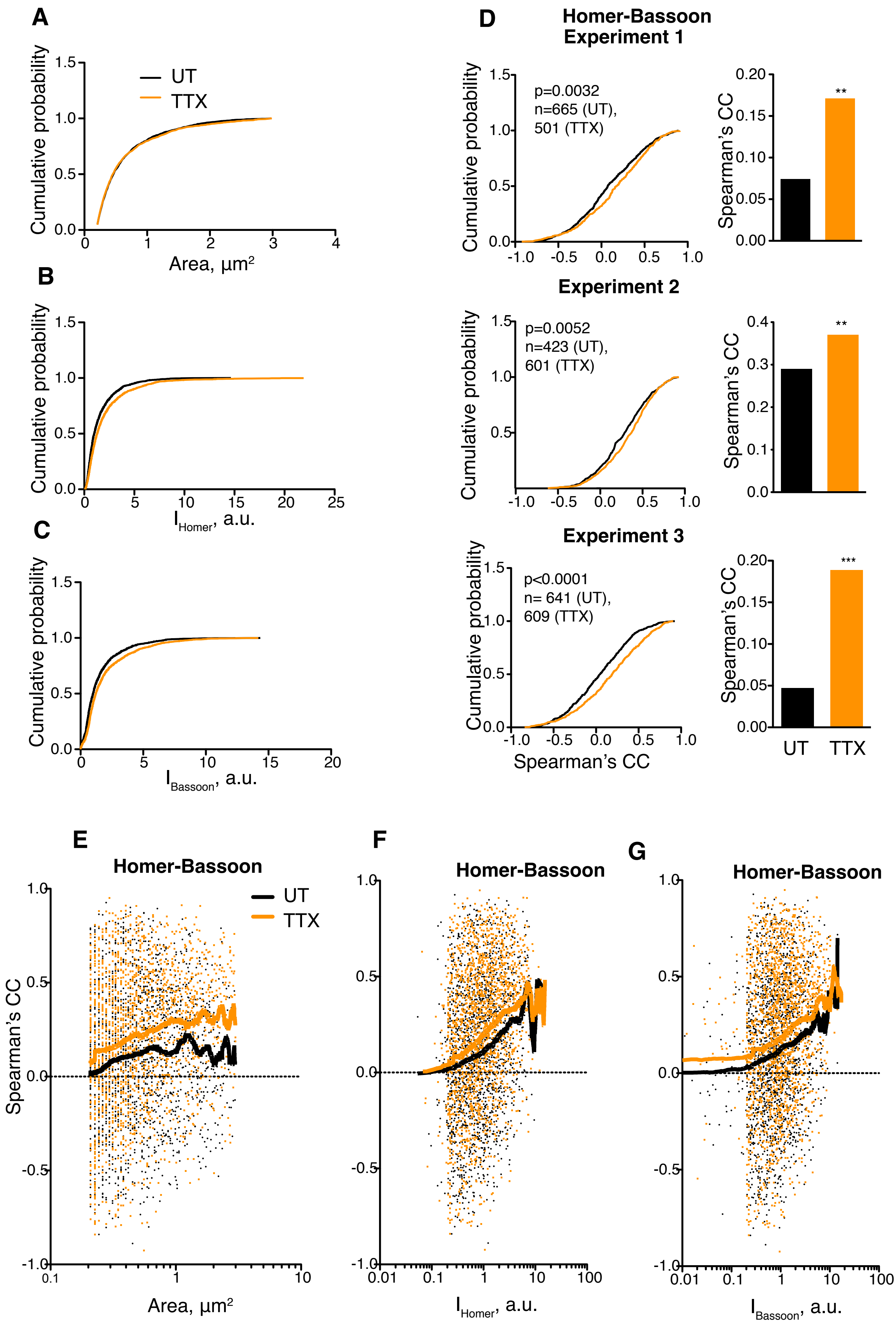

Supplementary Figure 2

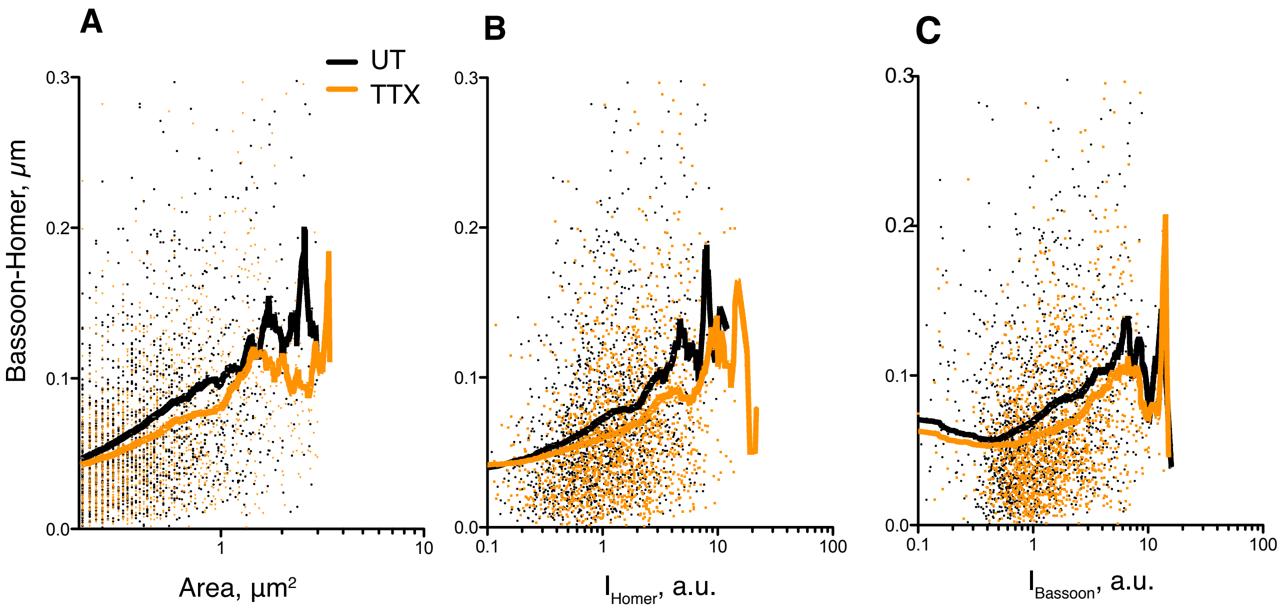

Supplementary Figure 3

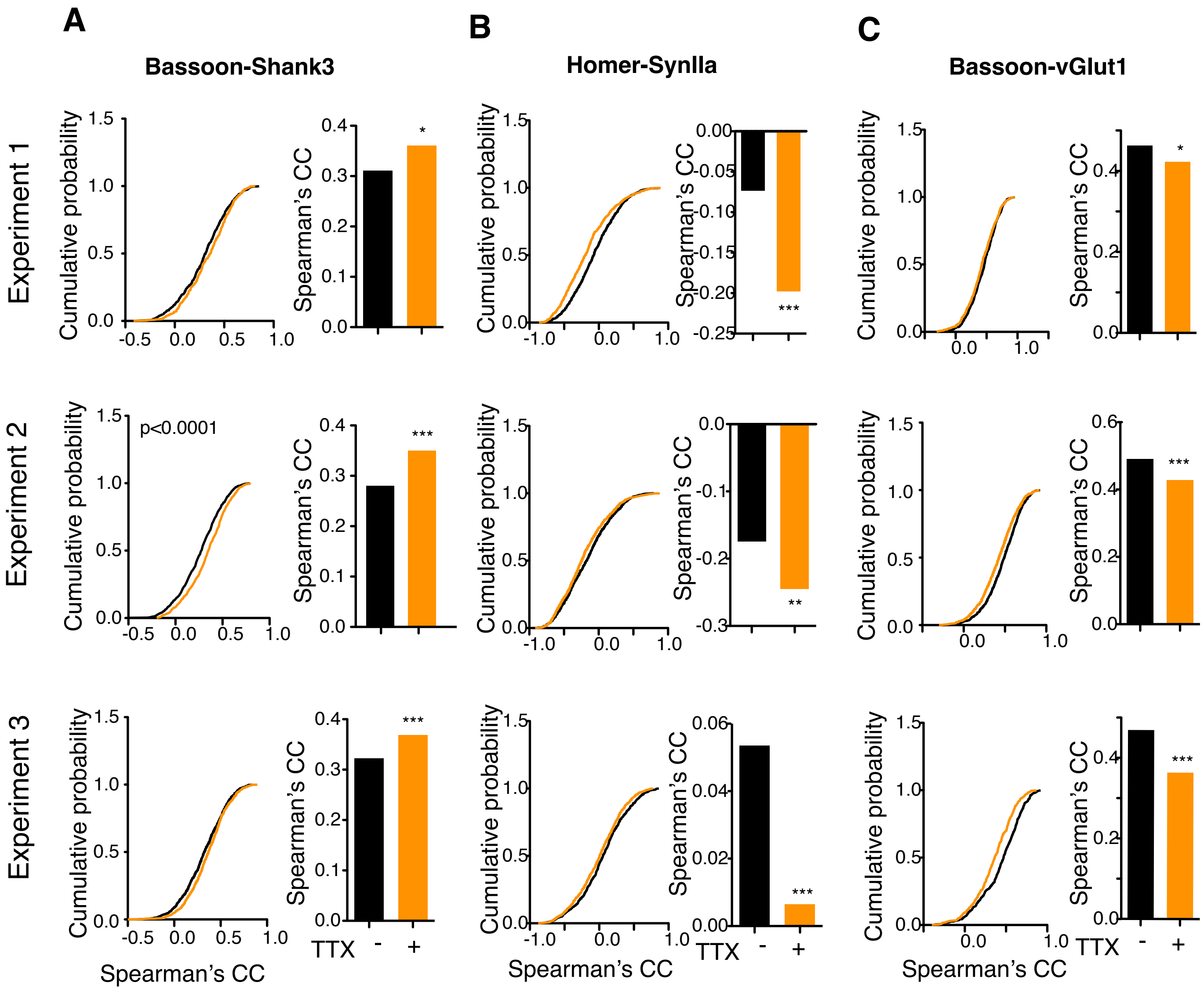

Supplementary Figure 4

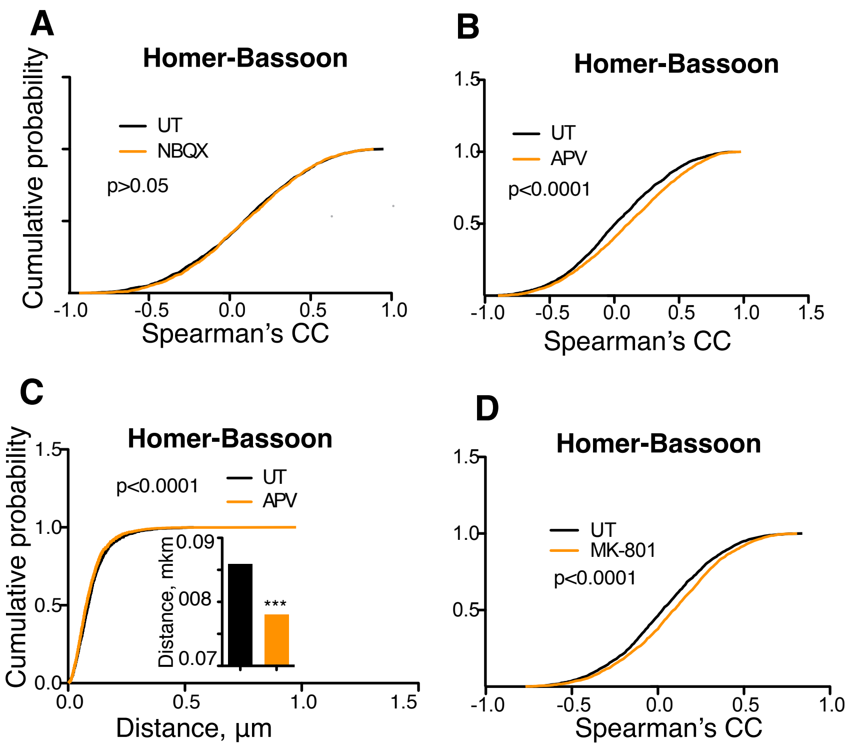

Supplementary Figure 5

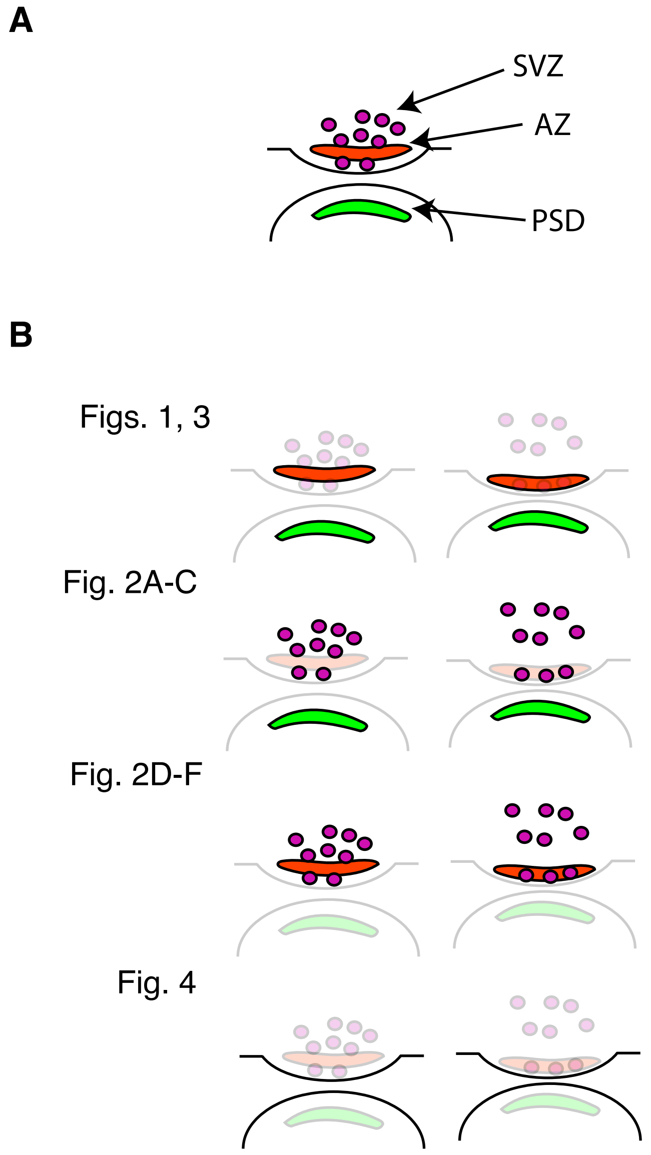

Supplement: Supplementary Information [file srep22703-s1.pdf]
